# Exploring Responsible Research and Innovation (RRI) in youth mental health: reflections from researchers and young people

**DOI:** 10.1186/s40900-026-00848-x

**Published:** 2026-02-06

**Authors:** Josimar Antônio de Alcântara Mendes, Mathijs Lucassen, Sarah Doherty, Ayan Mahamud, Carolyn Ten Holter, Chris Greenhalgh, Ellen Townsend, Chris Hollis, Marina Jirotka

**Affiliations:** 1 Responsible Technology Institute, Department of Computer Science, University of Oxford, 39a St. Giles, Oxford, OX1 3LW UK; 2 City St George‘s, University of London, Northampton Square, London, EC1V 0HB UK; 3 Sprouting Minds (Young People Advisory Group – Digital Youth Programme), University of Nottingham, Nottingham, NG7 2UH UK; 4 University of Nottingham, Nottingham, NG7 2UH UK

**Keywords:** Responsible research and innovation, Mental health, Youth, Young people, Coproduction, Participatory

## Abstract

**Background:**

Responsible Research and Innovation (RRI) promotes inclusive, anticipatory, and reflexive research practices that respond to societal needs. While widely applied in technological fields, its application in youth mental health remains limited. This study aimed to explore how RRI principles are understood and enacted within a large interdisciplinary programme on digital youth mental health in the United Kingdom, focusing on the perspectives of both researchers and young people.

**Methods:**

An online survey was conducted with 21 researchers and 5 young people (mean age = 21 years, standard deviation = 2.74) involved in the programme. The survey included open-ended questions exploring knowledge, attitudes, and practices related to RRI and youth mental health. Responses were analysed using Reflexive Thematic Analysis to identify patterns of meaning across the dataset and to generate themes.

**Results:**

Six themes were developed, reflecting participants’ knowledge, attitudes, and practices. Both researchers and young people conceptualised youth mental health as multifaceted, shaped by personal, social, and cultural factors, and existing along a continuum from flourishing to struggling. Young people highlighted digital harms and economic precarity, while researchers emphasised biopsychosocial determinants, offering complementary perspectives. Involving young people was seen as essential for challenging adult assumptions, improving clarity and relevance of tools, and strengthening ethical integrity. Barriers included communication gaps, entrenched hierarchies, inconsistent involvement, and the resource-intensive nature of participation. Key facilitators included mutual respect, care, flexibility, and procedural structures such as youth co-chairs (i.e., a young person co-leading the project/grant with the principal investigator/s) and regular collaborative meetings. Together, these elements demonstrated how RRI values can be embedded to foster meaningful and equitable youth–researcher partnerships.

**Conclusions:**

This study shows that applying RRI in youth mental health research enhances co-production by integrating diverse perspectives, addressing ethical concerns, and strengthening the quality and social relevance of research. To fully realise this potential, RRI must be embedded as an ongoing practice supported by intentional infrastructures, such as youth leadership roles, communication training, and opportunities for intergenerational dialogue. Crucially, funders must recognise and resource the relational, iterative, and time-intensive nature of responsible youth involvement. Embedding RRI in this field provides a valuable framework for moving beyond tokenistic consultation towards inclusive, future-oriented, and ethically grounded research.

**Supplementary Information:**

The online version contains supplementary material available at 10.1186/s40900-026-00848-x.

## Abstract (plain language summary)

This study looked at how to do research with young people, not just about them, in the area of youth mental health. We used the idea of “Responsible Research and Innovation” (RRI), which means planning ahead, listening carefully, and acting responsibly so that research is useful, fair, and safe for the people it affects. We asked researchers and young people who were working together to share their views and experiences. Both groups said youth mental health is complex and sits on a spectrum from doing well to struggling, shaped by personal factors (like sleep, mood, and coping) and social factors (like family, friends, money, and online life). Young people said their involvement helped challenge adult researchers’ assumptions and made tools and studies clearer, more relevant, and easier to use. They also pointed out practical needs for good partnerships: mutual respect, plain language, quick feedback, and real influence on decisions. Risks were flagged too – discussions can be emotionally tough, and privacy must be protected. Working together is rewarding but demanding. Jargon, time pressures, and power imbalances can get in the way. To fix this, the study suggests: involve young people from the very start; create roles like a Youth Co-chair; provide training to support good communication across ages; and bring young people and policymakers together for regular roundtables. We also call on funders to back this work properly, with time, flexibility, and resources. Done well, RRI helps produce safer, fairer, and more effective research for young people’s mental health.

**Supplementary Information:**

The online version contains supplementary material available at 10.1186/s40900-026-00848-x.

## Background

Responsible Research and Innovation (RRI) seeks to align research with societal values, needs, and expectations through inclusive, reflexive, and responsive approaches from the outset. It addresses not only scientific practice but also the broader societal impacts of innovation, particularly in emerging technologies [1, 2]. Over the past two decades, RRI has gained influence in policy and academia, promoting ethically grounded research that contributes meaningfully to societal well-being [3, 4].

Since the early 2000s, RRI has been emphasised, beginning with the USA’s ‘*21st Century Nanotechnology Research and Development Act*’ (2003) and the EU’s 2004 strategy on nanotechnology [5]. While initially addressing risks and subsequent mitigation strategies in fields like nanotechnology, geoengineering, and synthetic biology [5], RRI has since expanded to areas such as Information and Communication Technology (ICT), and robotics [6]. As RRI has travelled across domains, its concepts have increasingly been taken up beyond the Science, Technology, Engineering and Mathematics (STEM) fields in which it first gained prominence. For instance, RRI-related ideas have begun to inform work in psychology, including youth mental health^1^, particularly where researchers adopt co-produced and participatory approaches in digital youth mental health (e.g., participatory case-study methodologies for self-harm app development and co-produced qualitative analyses with young people) [7, 8]. Building on this emerging body of work on responsible participation in youth mental health projects, we focus here specifically on youth mental health as a field in which RRI principles are only starting to be explicitly named and systematically evaluated – for further detail, see Mendes et al.’s [8] scoping review, which addresses this trend in more depth and provides a dedicated discussion. However, this growing interest also brings a central challenge to the fore: despite its increasing uptake, RRI remains variably defined and interpreted across contexts, which has implications for how it can be applied, communicated and appraised in youth mental health research.

RRI lacks a universally agreed definition [1]. This reflects its adaptive nature, as RRI is shaped by project-specific needs and contextual factors, similarly to Patient and Public Involvement (PPI) [8]. Nonetheless, RRI retains a core set of values and principles, including anticipation, reflexivity, inclusiveness, and responsiveness [2, 5, 9, 10]. It promotes early stakeholder engagement and ethical reflection, embedding responsibility as integral to good scientific practice, not as an add-on [3]. RRI is also framed as a transparent, collaborative process aimed at producing ethically acceptable, sustainable, and socially desirable outcomes [1], spanning from design through to policy [11]. Van den Hoven [12] adds that responsible innovation must involve stakeholder engagement, risk anticipation, and governance mechanisms that honour diverse societal values.

Although RRI principles have been predominantly applied within STEM fields, their integration into youth mental health research remains limited [8] – which further strengthens the rationale and the focus for this paper. This gap may reflect the long-standing clinical orientation of adolescent research, the dominance of frameworks such as PPI, and limited clarity regarding how RRI diverges from or complements existing models [13]. It may also be attributed to the absence of RRI-specific mandates from funders [13]. In the United Kingdom (UK), most medical research, including the programme examined in this study, requires the implementation of PPI, a well-established framework focused on engaging patients and the public throughout the research process [8]. While PPI is essential and widely practised within healthcare settings, RRI offers a broader, anticipatory, and systemic approach that extends beyond immediate service-user engagement. RRI encourages early-stage inclusion of a wider set of stakeholders (e.g., policymakers, regulators, practitioners), fosters anticipation of societal and ethical implications throughout the innovation lifecycle, and embeds continuous reflexivity and governance into research systems [13, 14]. Emerging interdisciplinary initiatives, particularly in digital mental health, demonstrate that RRI not only complements but strengthens ethical rigour in PPI by embedding anticipatory reflection, governance, and broader stakeholder accountability into participatory practices [7, 8, 15] – for a more detailed discussion regarding the exercise of PPI and RRI in the realm ‘participatory approaches’ and youth mental health, see Mendes et al.’s scoping review [8].

Participatory approaches are increasingly seen as good practice in youth mental health research, especially in efforts to promote youth resilience [7, 16–20]. These approaches view young people as active contributors with ‘lived experience’ – that is, young people who have faced or are currently facing mental health struggles at some point in their lives. Integrating RRI into such work is a natural progression, aligning with its core principles of collaboration, inclusivity, and responsiveness [7, 13]. This alignment is particularly relevant given the ethical obligations prompted by the principle of the best interests of the child. This principle positions children and adolescents as rights-bearing individuals with agency, capable of participating both meaningfully and safely in decisions that affect them [21], as articulated in the United Nations’ (UN) Convention on the Rights of the Child [22], where the best interests obligation (Article 3) is coupled with children’s rights to be heard in all matters affecting them (Article 12) and to receive guidance consistent with their evolving capacities (Article 5). It also emphasises the importance of ensuring protective and supportive environments – an issue of heightened relevance in youth mental health research, where young people may be vulnerable or at risk [7, 8, 23]. Within this framework, provision, protection, and participation are understood as interdependent dimensions rather than competing priorities, with the UN’s Committee on the Rights of the Child explicitly describing the Convention as organised around these three ‘Ps’ [22]. Lundy’s [24] four-dimensional model of participation further translates Article 12 into the practical requirements of space, voice, audience, and influence, highlighting that children’s participation is only meaningful when they are given the opportunity to express views, supported to do so, listened to, and able to shape outcomes. Recent qualitative studies similarly show how staff, parents, and adolescents continually negotiate this balance in recovery-oriented inpatient care, with safety and security considerations needing to support, rather than automatically restrict, adolescents’ participation and emerging autonomy [25, 26].

These participation debates are also inherently epistemic. Research on epistemic injustice demonstrates that children and young people are frequently given reduced credibility as ‘knowers’ and excluded from shaping the interpretive frameworks that structure welfare, education and mental health systems [27, 28]. It is also known that stigma, institutional cultures, and intersecting inequalities can lead to young people’s accounts of distress, recovery, and family life being discounted or misinterpreted, including in psychiatric and mental health contexts [29, 30]. Attending explicitly to these epistemic hierarchies and forms of injustice is therefore essential if RRI-aligned participatory approaches are to move beyond tokenism and to truly acknowledge children’s rights to be heard and to influence decisions that affect them [21, 22, 27]. Consequently, adopting RRI principles in this context is not only methodologically sound but ethically imperative, offering a framework through which researchers can balance innovation with care, empowerment with safeguarding, and participation with protection.

Moreover, the rise of digital mental health tools introduces challenges that RRI is well-equipped to tackle. RRI encourages researchers to anticipate risks like emotional distress and data privacy concerns, embedding safeguards into intervention design while promoting reflection on exclusion and equity [7]. Yet, RRI remains underused in youth mental health research. Even with participatory methods like co-research and co-design, RRI principles are seldom fully applied [8]. This underscores the need for empirical research on how RRI is understood and enacted in youth mental health research. This study was designed to help address this gap. It explores the implementation of RRI principles within a UK-based, interdisciplinary digital youth mental health research programme spanning multiple universities and interlinked work packages, which examined how digital environments shape young people’s mental health and how responsible, participatory approaches can be embedded across both research and future innovations. The programme involved both researchers and young people through a range of participation structures (e.g., advisory and leadership roles, and work package-specific engagement), providing a relevant context to examine how RRI is interpreted and enacted in practice. For more information, check https://digitalyouth.ac.uk/ and the additional information provided in the ‘Participants’ subsection of the Methods. Drawing on data from both researchers and young people, this study examines how RRI principles are interpreted and enacted in practice. By capturing participants’ views on youth mental health and collaboration efforts, the study clarifies how RRI can support more ethical, inclusive, and effective partnerships, with the intention of ultimately strengthening research methodologies in this field.

## Methods

This study draws on an online survey with closed- and open-ended questions designed to explore researchers’ and young people’s knowledge, attitudes, and practices regarding RRI. We applied the Knowledge, Attitudes and Practices (KAP) model as an organising heuristic to structure both the survey content and the subsequent interpretation of the qualitative data, given its versatility across quantitative, qualitative, and mixed-method designs [31, 32]. Widely used in health research, it helps evaluate adherence to protocols, identify key issues, and inform suitable interventions, as well as to explore how different forms of knowledge relate to interpretations, values, and behaviours in specific contexts [31, 33].

The ‘Knowledge’ dimension refers to what the participant knows or understands about a particular subject [34, 35]. In the context of public health, this could include facts about concepts such as mental health, disease prevention, or about available interventions or forms of treatment. In this study, ‘Knowledge’ was operationalised as participants’ conceptual and experiential understandings of RRI and youth mental health (for example, how they defined these constructs, how they understood their determinants, and how they located their own role in relation to them), rather than as factual recall or literacy alone. ‘Attitudes’ refers to the participant’s feelings, beliefs, and values towards the particular subject – it is about how they perceive the issue and their emotional or cognitive orientation towards it [36, 37]. For example, a young person may understand that persistent low mood, anxiety, or self-harm ideation warrants professional support but hold negative attitudes towards seeking help (for instance, due to stigma, mistrust, or fear of burdening others). ‘Practices’ refers to KAP’s behavioural dimension and addresses the actions people take. It is the observable behaviour that results from a combination of their knowledge and attitudes [34, 35, 38]. For instance, a young person’s use of coping strategies (such as talking to a trusted adult or using a digital mental health resource) or engagement with support services will reflect both their knowledge about mental health difficulties and their attitudes towards seeking help and support. Consistent with contemporary uses of the KAP framework, we treated these three dimensions as analytically distinct but interdependent, with knowledge providing a foundation for, rather than a deterministic cause of, subsequent attitudes and practices [33].

### Participants

A total of 26 participants took part in this study, comprising 21 researchers and 5 young people. As shown in Table 1, the majority of the youth participants were female, with a mean age of 21 years (SD = 2.74). Most had prior experience participating in mental health research projects.

Both researchers and young people were recruited via email invitations circulated across the programme’s work packages. The young people were involved in the programme at different levels of participation: transformative, intermediate, and descriptive [39, 40]. At the *transformative* level, they shared decision-making and reciprocal expertise, for example through the Young People’s Advisory Group (YPAG) and Youth Co-Chair^2^ roles that helped to shape programme priorities and decisions from the outset; at the *intermediate* level, they took part in active co-development and championing of research materials with more limited decision-sharing; and at the *descriptive* level, they contributed through consultation and feedback at specific stages across different work packages. Safeguarding processes, including risk assessment, signposting to support, and agreed pathways for responding to distress, were implemented within each work package in line with local ethics approvals and good-practice guidance on PPI with young people in mental health research. These procedures were tailored both to the specific focus of each work package and to the level of young people’s involvement, with concrete examples of this approach provided elsewhere for the Digital Youth programme (for an illustration, see Babbage et al. [7]).

### Survey design and data collection

Data were collected between February and May 2024 in the UK via an online platform: Qualtrics. Researcher participants included senior academics (e.g., full professors) to early career researchers (e.g., research assistants), with most holding doctoral degrees. The young people were university students or recent graduates with lived experience (i.e., any kind of ‘mental health struggle’ – for an example and further details, see Babbage et al. [7]). The programme investigated the interface between digital environments and youth mental health, comprising eight interlinked work packages, including a cross-cutting strand focused on embedding responsible research practices.

The survey comprised items organised into sections: sociodemographic information; knowledge, attitudes, and practices related to RRI; conceptualisations of mental health; and experiences of collaboration between young people and researchers within the programme. The KAP-related questions were adapted from the RRI Prompts and Practice Cards [41], ensuring that prompts aligned with the AREA framework (Anticipate, Reflect, Engage, Act) dimensions of RRI [41]. This design aimed to generate data on both the theoretical awareness and applied integration of RRI values amongst participants. In this paper, we report upon a Reflexive Thematic Analysis of the participants’ responses to the open-ended questions – see Additional File 1.

### Ethical considerations

This study and its materials (e.g., information sheet and consent form) were approved by the University of Oxford’s Computer Science Departmental Research Ethics Committee (Ref: CS_C1A_23_033). Given the sensitive nature of this study, participants opted not to permit the release of full transcripts in a public repository. The processes of data anonymisation and the maintenance of data integrity were consistent with good practice in adolescent and youth mental health [42–44]. Nonetheless, anonymised excerpts and relevant supporting materials are accessible in the Additional Files.

**Table 1:** Young people’s background and experience

Young people ID	Age	Gender	How many work packages do you collaborate with within the programme?^a^	Have you been involved in a Mental Health Project before?
YP1	22	Female	2	Yes, 2 projects
YP2	21	Female	0^b^	Yes, 2 projects
YP3	25	Male	4	Yes, 5 projects or more
YP4	19	Female	1	No, this is my first time
YP5	18	Male	1	Yes, 1 project

^a^The programme has a total of eight work packages

^b^Some young people did not work directly with an individual work package, as they were part of a broader YPAG

Amongst the researchers, 62% (*n* = 13) were women. As detailed in Table 2, most participants had extensive experience in their respective fields, especially in relation to mental health. Notably, of the 21 researchers, the largest proportion (57%, *n* = 12) had a background in Psychology. Additionally, 19% (*n* = 4) held degrees in Health Sciences and Biomedical Engineering, while 10% (*n* = 2) each had expertise in Computer Science and Information Systems/Technology. Overall, the sample included early career (e.g., a research assistant) and mid-career researchers (e.g., research fellows) as well as senior researchers (e.g., professors).

**Table 2:** Researcher participants’ background and experience

Researcher ID	Main Degree	Role	Experience with Mental Health (Years)
R1	Health Sciences	Co-investigator	More than 10 years
R2	Psychology	Co-investigator	More than 10 years
R3	Computer Science	Co-investigator	3 to 4 years
R4	Psychology	Co-investigator	9 to 10 years
R5	Psychology	Co-investigator	7 to 8 years
R6	Sociology	Post-doctoral Researcher	3 to 4 years
R7	Psychology	Co-investigator	More than 10 years
R8	Information Systems/ Technology	Co-investigator	9 to 10 years
R9	Information Systems/ Technology	Research Fellow	1 to 2 years
R10	Psychology	Co-investigator	7 to 8 years
R11	Biomedical Engineering	Research Fellow	1 to 2 years
R12	Psychology	Research Fellow	More than 10 years
R13	Health Sciences	Research Assistant	1 to 2 years
R14	Epidemiology	Research Fellow	5 to 6 years
R15	Psychology	Post-doctoral Researcher	7 to 8 years
R16	Psychology	Co-investigator	More than 10 years
R17	Psychology	Project Manager / Admin Staff	5 to 6 years
R18	Psychology	Co-investigator	More than 10 years
R19	Computer Science	Co-investigator	7 to 8 years
R20	Psychology	Co-investigator	More than 10 years
R21	Psychology	Research Fellow	7 to 8 years

In terms of researchers’ roles within the sample, 57% (*n* = 12) of the researchers served as co-investigators, and 33% (*n* = 7) were research fellows or post-doctoral researchers. The majority of them (67%, *n* = 14) had more than 7 years of experience within the field of mental health research, with 9.5% (*n* = 7) having over 10 years of experience.

### Data analysis

**Fig. 1:**
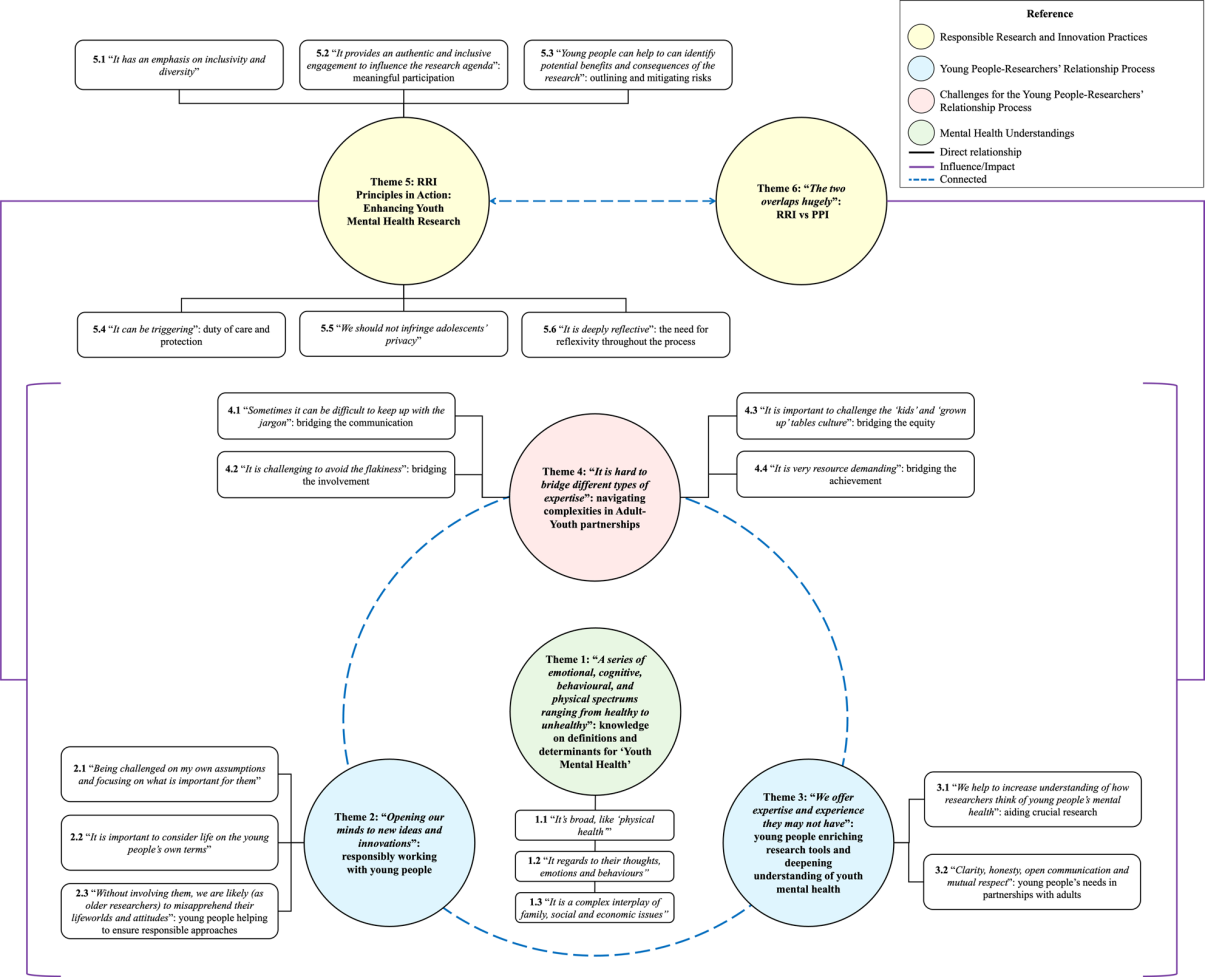
Thematic map

All data were analysed using Reflexive Thematic Analysis (RTA), which identifies and interprets patterns in qualitative data while recognising researcher subjectivity and socio-cultural context [45–47]. The first author conducted the data analysis following the six phases of RTA [45, 46], drawing on guidance from Nowell et al. [48] and Mendes et al. [49]:

- *familiarisation*: the first author reviewed all responses to grasp the dataset as a whole, taking notes of initial ideas and identifying potential emerging patterns;
- *first level of analysis (open coding)*: using NVivo 14 (version 14.24.0) for MacBook M1, the data were openly coded and categorised according to the study’s objectives. Twelve codes were generated, grounded in the data (see Additional File 2);
- *second level of analysis*,* generating initial themes*: codes were synthesised into 9 Candidate Themes and 23 Features (i.e., subthemes), representing meaningful patterns (see Additional File 3);
- *reviewing and defining themes*: candidate themes and features were thoroughly examined and refined to ensure they were coherent and distinct. This phase resulted in 6 Final Themes and 18 Features;
- *anchoring themes and thematic map*: each theme was linked to specific participants to ensure the dependability of the results, rather than quantifying the themes based on ‘participant frequency’. Additionally, a thematic map was developed to illustrate interrelations between themes – see Fig. 1;
- *ensuring trustworthiness*: to strengthen the trustworthiness of the results, via credibility and dependability checking [48, 50], we engaged in ‘member reflections’ [45, 46]. The themes were initially reviewed by members of the project’s YPAG, who are the third and fourth authors of this paper. They created a young-people-friendly version of the results in the form of a video clip (https://bit.ly/3G2sNl4*)* and, with support from the first author, facilitated an online discussion based on the video with eight other YPAG members (seven female, one male; M = 26.4 years, SD = 2.58). Their reflections, insights, and feedback were incorporated into the analysis and are summarised in the subsection ‘*YPAG’s Reflections on the Results*’. This iterative and participatory process involving the programme’s Young People’s Advisory Group (YPAG) was central to the study’s approach, reinforcing the commitment to embedding young people’s perspectives throughout the research in line with RRI principles of inclusion, responsiveness, and reflexivity – for further details, please see Mendes et al. [51]. Their input provided youth-validated interpretations that enhanced the authenticity and relevance of the findings, as they contributed meaningfully to the conceptualisation, analysis, and dissemination of research [52–54].

A reflexivity process was maintained throughout the data analysis process, with continuous reflection on how the researcher’s background and perspectives might influence the analysis [55, 56]. The reflexivity statement of the first author (leading the analysis) is available in Additional File 4.

## Results

Throughout this section, participants are identified as ‘R’ (Researchers) or ‘YP’ (Young People), followed by a participant number (e.g., R1, YP1), as shown in Table 2. Table 3 outlines the themes and their features, anchored in participants’ accounts.^3^ Rather than quantifying themes, this anchoring highlights the dynamic interplay between researchers’ and participants’ perspectives, enhancing transparency and trustworthiness [49].

**Table 3:** Themes and features generated by the Reflexive Thematic Analysis and their anchoring on the data

Theme	Anchoring	KAP Dimension
**Theme 1: ** ***“A series of emotional, cognitive, behavioural, and physical spectrums ranging from healthy to unhealthy”*** **: Knowledge on Definitions and Determinants for ‘Youth Mental Health’**		
**Feature 1.1 –** “*It’s broad*,* like ‘physical health’*”	R1, R3, R4, R11, R12, R15, R17, R20, YP1, YP2	Knowledge
**Feature 1.2 –** “*It regards to their thoughts*,* emotions and behaviours*”	R1, R3, R5, R6, R7, R9, R10, R11, R12, R13, R15, R16, R17, R20, YP1, YP2, YP3, YP4, YP5	
**Feature 1.3 –** “*It is a complex interplay of family*,* social and economic issues*”	R1, R2, R4, R5, R6, R8, R9, R10, R11, R15, R17, R20, YP1, YP2, YP4, YP5	
**Theme 2: “** ***Opening our minds to new ideas and innovations*** **”: Responsibly Working with Young People**		
**Feature 2.1 –** “*Being challenged on my own assumptions and focusing on what is important for them*”	R1, R2, R8, R14, R17, R18, R21	Attitudes
**Feature 2.2 –** “*It is important to consider life on the young people’s own terms*”	R2, R3, R5, R9, R12, R13, R18	Attitudes
**Feature 2.3 –** “*Without involving them*,* we are likely (as older researchers) to misapprehend their lifeworlds and attitudes*”: young people helping to ensure responsible approaches	R2, R3, R4, R5, R7, R15, R16, R17, R18, R20, R21	Practices
**Theme 3: “** ***We offer expertise and experience they may not have*** **”: Young People Enriching Research Tools and Deepening Understanding of Youth Mental Health**		
**Feature 3.1 –** “*We help to increase understanding of how researchers think of young people’s mental health*”: aiding crucial research	YP1, YP2, YP4	Practices
**Feature 3.2 –** “*Clarity*,* honesty*,* open communication and mutual respect*”: young people’s needs in partnerships with adults	YP1, YP2, YP4, YP5	Attitudes
**Theme 4: “** ***It is hard to bridge different types of expertise*** **”: Navigating Complexities in Adult-Youth Partnerships**		
**Feature 4.1 –** “*Sometimes it can be difficult to keep up with the jargon*”: bridging the communication	R5, R12, R15, R16, R17, R18, R20, R21, YP2, YP4	Attitudes and Practices
**Feature 4.2 –** “*It is challenging to avoid the flakiness*”: bridging the involvement	R2, R3, R4, R9, R13, R15	Practices
**Feature 4.3 –** “*It is important to challenge the ‘kids’ and ‘grown-up’ tables culture*”: bridging the equity	R17, YP2, YP5	Attitudes
**Feature 4.4 –** “*It is very resource demanding*”: bridging the achievement	R4, R8, R9, R21	Practices
**Theme 5: RRI Principles in Action: Enhancing Youth Mental Health Research**		
**Feature 5.1 –** “*It has an emphasis on inclusivity and diversity*”	R1, R4, R8, R17, YP1, YP4	Practices
**Feature 5.2 –** “*It provides an authentic and inclusive engagement to influence the research agenda*”: meaningful participation	R1, R2, R3, R10, R11, R16, R18, R21, YP2	Attitudes and Practices
**Feature 5.3 –** “*Young people can help to can identify potential benefits and consequences of the research*”: outlining and mitigating risks	R2, R8, R9, R12, R15, R16, YP5	Knowledge and Practices
**Feature 5.4 –** “*It can be triggering*”: duty of care and protection	R1, R2, R3, R4, R8, R11, R12, R13, R16, R17, YP5	Attitudes and Practices
**Feature 5.5 –** “*We should not infringe adolescents’ privacy*”	R9, R15, R18	Attitudes and Practices
**Feature 5.6 –** “*It is deeply reflective*”: the need for reflexivity throughout the process	R2, R3, R4, R5, R18, R20, R21	Knowledge and Practices
**Theme 6: “** ***The two overlap hugely*** **”: RRI vs. PPI**	R1, R2, R3, R4, R5, R8, R11, R12, R13, R15, R16, R17, R21	Knowledge and Practices

As illustrated in Fig. 1, the themes offer insights into how researchers and young people co-construct the meaning of youth mental health and navigate the complexities of collaboration. Taken together, the themes generated resonate strongly with foundational RRI principles, particularly concerning inclusivity, responsiveness, reflexivity, and anticipation [1–3].

### Theme 1: “*A series of emotional, cognitive, behavioural, and physical spectrums ranging from healthy to unhealthy*”: knowledge on definitions and determinants for ‘Youth mental health’

This theme explores how researchers and young people define youth mental health, highlighting its physical, cognitive, psychological, and emotional dimensions. It also includes family, social, and economic factors, underscoring the complex interplay of determinants. Participants’ definitions reflect a shared view of mental health as existing on a continuum from healthy to unhealthy.

Feature 1.1 (“*it is broad*,* like ‘physical health’*”) illustrates the wide-ranging and multifaceted nature of mental health, drawing parallels to physical health. As one researcher noted, mental health “*is broad*,* like ‘physical health’ - it captures a person’s wider psycho-social well-being and is more than the absence of disease*” (R1). This understanding was echoed by another researcher who emphasised that “*like physical health*,* we all have mental health*,* and it is something that we need to take care of as we do our physical selves*” (R12). A young person also linked mental health to physical well-being: “[it is related to] *physical well-being*” (YP1). The determinants of mental health highlighted under this feature further underscore its complexity. Biological factors, such as a genetic predisposition to mental ill-health: “*Many factors affect the development and maintenance of adolescent mental health*,* including biological factors such as genetic predisposition*” (R17), as well as lifestyle-related elements like sleep, diet, and nutrition (R20, R3), were all seen as influencing an adolescent’s mental health. A young person also mentioned the impact of puberty on mental health (YP2).

Feature 1.2 (“*it regards to their thoughts*,* emotions and behaviours*”), this focuses on the psychological and emotional dimensions of mental health. Several participants, both researchers and young people, described mental health as being related to one’s thoughts, emotions and behaviours as these apply to their functioning in everyday life (R3, R5, R6, R10, R12, R15, R16, R17, R20, Y1, YP2, YP4, YP5). For example, one researcher defined youth mental health as an individual’s “*health with regard to their thoughts*,* emotions and behaviours*” (R17). This perspective was echoed by a young person who referred to mental health as related to “*the quality/health of your emotional*,* psychological well-being*” (YP1). Another young person similarly referred that “[mental health is] *a state of mental well-being that enables people to cope with the stresses of life*” (YP3). The determinants pointed out were primarily psychological, with a focus on factors such as coping strategies, emotion regulation, and cognitive flexibility (R1, R20). Other influences cited included individual temperament, personality, neurotype, trauma experiences and external pressures, like academic demands (R5, YP2, YP4).

Feature 1.3 (“*it is a complex interplay of family, social and economic issues*”) emphasises the social aspects related to mental health, which is seen as “*a complex interplay of constitutional risk and proximal social environmental exposures*” (R2), particularly at the family and peer level. This idea is reinforced by a young person who linked mental health to social well-being (YP1). The determinants identified here include social factors such as “*having strong connections with others*” (R15), relationships with family and peers, as well as potential challenges such as poverty and for some marginalisation (R1, R12, YP). Researchers also pointed to the significance of broader societal influences, including a person’s socioeconomic status, housing situation, and overall safety (R3, R18, R20). Young people similarly highlighted the importance of factors such as “*socioeconomic factors, community* [bonds]” (YP1), “[issues related to] *class, race, gender*” (YP4) and family dynamics, digital activity and possible exposure to violence in shaping their mental health (YP2, YP5).

Overall, this theme presents a multidimensional knowledge of youth mental health. It recognises its formation through a complex interaction of physical, emotional, social, and economic factors, and frames it as a continuum from healthy to unhealthy states.

### Theme 2: “*Opening our minds to new ideas and innovations*”: responsibly working with young people

This theme highlights researchers’ recognition of the value of incorporating young people’s perspectives into youth mental health projects. Engaging young people challenges assumptions, aligns research with their priorities, and empowers them to contribute meaningfully. This approach helps ensure that interventions are relevant, impactful, and grounded in lived experience.

Feature 2.1 (“*being challenged on my own assumptions and focusing on what is important for them*”) reveals how researchers benefit from being challenged by young people. They noted that involving young people often leads to new insights and helpfully disrupts existing assumptions, which assists in keeping research aligned with young people’s priorities. For instance, one researcher remarked that young people can make researchers “*more willing to open our minds to new ideas and innovations*” (R21). Another highlighted that collaboration with young people aids in “*avoiding adult impositions or inappropriate assumptions and blind spots in defining the problem*” (R18) and provides “*fresh insights that would otherwise not be present*” (R14). Researchers also acknowledge that collaboration with young people helps them to get “*the design right – i.e.*,* avoiding off-putting language or presentation*,* getting the best ideas about approach and context of use*,* help in brainstorming downsides and opportunities*” (R8) and that “*it is really refreshing to hear the young people’s perspective on the issues*,* and often be challenged on my own assumptions*” (R2). This approach is crucial not only for refining project designs but also for ensuring they are valuable to the intended end users: “*there are many strengths* [in the collaboration with young people], *mostly it is to see if the project is likely to be valuable to young people*” (R1) as we “*can’t assume the views of older PIs* [Principal Investigators] *are a perfect reflection of theirs* [young people]” (R8).

Feature 2.2 (“*it is important to consider life on the young people’s own terms*”) underscores the necessity of aligning research with young people’s lived experiences. Researchers stressed the importance of understanding and incorporating young people’s perspectives to ensure interventions are effective and relevant (R5, R9, R12, R13). For example, one researcher emphasised that it is “*important to consider life on the young people’s own terms when identifying potential consequences for the intervention or tool under development*” (R3). Others highlighted that involving young people in the project offers valuable insights into their perspectives on the tools developed for them: “*involving young people in the project would aid in gaining insights into their perspective regarding the tools developed for them*” (R9), “*without having young people on board*,* we will not know if the research is being conducted in a way that is meaningful to them*” (R12). Additionally, researchers reinforced that young people’s involvement aids the research process: “[young people help us] *developing methods*,* interpreting findings and designing impact/outcomes*” (R18).

Feature 2.3 (“*without involving them, we are likely (as older researchers) to misapprehend their lifeworlds and attitudes*”: *young people helping to ensure responsible approaches*”) addresses the risks associated with not involving young people in the research process. Researchers noted that excluding young people can lead to misunderstandings of their needs and attitudes, potentially resulting in ineffective or unused tools and interventions (R2, R3, R15, R16, R17, R20). For instance, one researcher highlighted that “*the intended beneficiaries are young people and… *[not involving them could result in]* unwanted/unused technologies*” (R3). Another added that young people’s involvement is crucial to “*ensure that the research and projects are rooted in actual realities of lives of young people and therefore are more likely to be effective*” (R17). Additionally, the involvement of young people “[can help researchers to] *have a better understanding or awareness of the data which is used to influence policy*” (R5) as working with them can provide “*essential insight to ensure the work is meaningful/ likely to lead to benefits*” (R7), making “*research questions and themes more relevant and impactful*” (R4).

In conclusion, this theme highlights the value of integrating young people’s perspectives into youth mental health projects. Challenging assumptions and involving them meaningfully enhances both the relevance of interventions and alignment with RRI principles such as inclusivity, responsiveness, and ethical collaboration.

### Theme 3: “*We offer expertise and experience they may not have*”: young people enriching research tools and deepening understanding of youth mental health

This theme highlights young people’s contributions to youth mental health projects, showing how their insights and expertise enrich research. It also underscores the need to support young people through respectful, meaningful collaboration.

Feature 3.1 (“*we help to increase understanding of how researchers think of young people’s mental health*”: *aiding crucial research*) captures how young people contribute to enhancing the research process. Their involvement is seen as essential in improving research tools and methodologies related to mental health (YP1, YP2, YP4). For instance, one young person noted that their participation helps to “*increase understanding of how researchers think of young people’s mental health*” (YP2) and aids “*crucial research in this area*,* offering expertise or experience they* [researchers] *may not have otherwise*” (YP1). They also highlighted that asking “*the opinions of adolescents and include them in relevant areas of research*,* for example in the design of questionnaires*” (YP2) can ensure that research tools are better suited to their needs.

Young people also signposted that the collaboration between them and researchers is a ‘two-way street’ as “*different points of view between young people and researchers cause discussions which lead to conclusions neither group would have necessarily considered on their own*” (YP4). Hence, this dynamic seems to be instrumental in refining research approaches and ensuring they are effectively tailored to address youth mental health concerns.

Feature 3.2 (“*clarity*,* honesty*,* open communication and mutual respect*”: *young people’s needs in partnerships with adults*) addresses the essential components of successful collaborations between young people and researchers. Young people emphasised the need for clarity, honesty, and open communication in these partnerships. For instance, one young person stressed that “*clarity*,* honesty*,* open communication and mutual respect*” (YP2) are critical for effective collaboration. Another highlighted the importance of researchers being “*open*,* understanding*,* and compromising*” (YP1) while ensuring that “*young people feel heard and important to the research and making the benefits of research clear*” (YP2). Respectful treatment was also a significant factor (YP4, YP5), with one young person noting a positive experience of feeling “*really valued*,* compared to my experience in the NHS or other services which are conducting PPI activities*” (YP5).

In conclusion, this theme emphasises the importance of involving young people in refining research tools and aligning methodologies with their needs. Mutual respect, clear communication, and recognition of contributions are key to effective collaboration and reflect core RRI principles of inclusivity, transparency, and responsiveness.

### Theme 4: “*It is hard to bridge different types of expertise*”: navigating complexities in adult-youth partnerships

This theme examines the challenges of Adult-Youth partnerships in youth mental health projects, including communication barriers, sustaining meaningful involvement, and ensuring equitable collaboration. It highlights the complexities of bridging different forms of expertise and experience between researchers and young people.

Feature 4.1 (“*sometimes it can be difficult to keep up with the jargon: bridging the communication*”) addresses the communication barriers that can arise between researchers and young people. One young person noted the challenge of understanding academic jargon, stating: “*sometimes it can be difficult to keep up with the jargon*” (YP4). Researchers also recognised this issue (R5, R18), with one noting that “*trying to explain complex research and academic terms in a way that is understandable to them* [young people] *is difficult and something I have always found challenging*” (R12). The difficulty in making research comprehensible for young people is evident, as researchers acknowledged the need to make their communication more accessible: “*I am so used to speaking and writing to an academic audience… I need to ensure I make what I say and write to young people more comprehensible*” (R12). It is also important to be “*careful not to assume young people know/understand concepts or words which are specific to research (or the professional world in general)*” (YP4).

Feature 4.2 (“*it is challenging to avoid the flakiness”: bridging the involvement*”) focuses on the difficulties of maintaining consistent engagement from young people. Researchers have noted various challenges, such as coping with perceived “*flakiness*” (R3), which involves dealing with slow and erratic responses, intermittent involvement, and conflicting life pressures - which makes it hard to ensure that their involvement would be “*subjectively worthwhile for them* [i.e., the young person]” (R3). One researcher observed that young people are “*remarkably busy and a bit cagey about participating*” (R9), and another noted that “*it’s been difficult to get them involved*” (R15). The need for consistent engagement is evident, but maintaining it proved to be a significant challenge highlighted by researchers: “*getting responses from young people are often difficult*” (R13) as much as their “*consistent engagement*” (R2).

Feature 4.3 (“*it is important to challenge the ‘kids’ and ‘grown-up’ tables culture*”: *bridging the equity*) highlights the importance of addressing power dynamics and hierarchies within these partnerships. Some young people observed that there is still a prevalent “*culture of* [a]*‘kids table’ for people with lived experience and ‘grown-up table’ for researchers*” (YP5). Participants emphasised the need to break “*down barriers in terms of hierarchies*,* language*” (R17), noting that this is essential for ensuring “*that the young people understand their role and the impact of what they are doing*” (YP2).

Feature 4.4 (“*it is very resource demanding*”: *bridging the achievement*) explores the resource-intensive nature of maintaining effective partnerships. Researchers reported that working with young people often requires significant resources and flexibility. Some of them highlighted the challenge of providing “*flexible ways for engagement that are compatible with office hours*” (R4) as “*it’s hard working flexibly to suit others but already working long hours*” (R21), indicating the need for adaptable engagement strategies. Another researcher noted that the process can be “*very resource demanding… and it can lead to exhaustion with trying to keep up*” (R21). Relatedly, “*finding a suitable time to approach young people*” (R9) was another barrier. In sum, there is “*a risk in doing RRI* [as you can] *become bogged down - wide consultation is an ‘expense’ in time (and somewhat in money)*” (R8).

In conclusion, this theme highlights the challenges of navigating Adult-Youth partnerships in youth mental health research. Communication gaps, uneven engagement, power dynamics, and resource constraints all add to the complexity of these collaborations.

### Theme 5: RRI principles in action: enhancing youth mental health research

This theme explores how RRI principles are applied in youth mental health research, highlighting their role in shaping youth involvement and responsible research practices. It emphasises inclusivity, meaningful engagement, risk management, and privacy as key to enhancing research quality and impact, as reflected in both researchers’ and young people’s perspectives.

Feature 5.1 (“*it has an emphasis on inclusivity and diversity*”) highlights the role of RRI in fostering a diverse and inclusive research approach. Young people recognise that RRI’s emphasis on inclusivity can enhance the diversity of participants involved in research. Some young people remarked: “[RRI provides] *an emphasis on inclusivity* [that] *could lead to a more diverse approach to involving young people in the research*” (YP4) by “*making sure as many different people can contribute*” (YP1). Researchers also acknowledge this benefit, noting that RRI encourages rigorous consideration of diverse stakeholder views and the creation of opportunities for broad participation. For example, some stated, “[RRI] *encourages a rigorous and thorough approach to considering and including the views of a wide range of stakeholders*” (R17) and also creates “*opportunities for young people to explore the possibilities associated with e-health*,* not just in trials*,* but in informing work packages and making meaningful changes*” (R1). This focus on inclusivity helps ensure that the research is relevant to a broad audience and reflects various perspectives as RRI fosters a “*wide stakeholder involvement to ensure opportunities and downsides are well-considered*” (R8).

Feature 5.2 (“*it provides an authentic and inclusive engagement to influence the research agenda”: meaningful participation*) underscores the importance of genuine and meaningful participation^4^ in shaping the research agenda. Researchers emphasise that RRI principles facilitate authentic engagement by requiring intentional and sustained efforts to involve young people in the research process. One researcher noted that “[an RRI approach provides an] *authentic and inclusive engagement to influence the research agenda*” (R10). This was also mentioned by another researcher who highlighted the necessity of “*involving affected youth in the co-design of research*,* impact and outreach*,* and critically interrogating proposed programmes or ‘solutions’ from multiple perspectives including children and young people*” (R18). Such involvement helps ensure that the research agenda aligns with the needs and concerns of young people, making the research more relevant and impactful, and ultimately more responsible: “[RRI] *protects and empowers the young people*,* and that has greater and more effective involvement of the young people*” (R11), “[RRI integrates] *the young people within research to further ensure that the research is actually relevant and beneficial to those with whom it affects*” (YP2).

Feature 5.3 (“*young people can help to identify potential benefits and consequences of the research*”: *outlining and mitigating risks*) focuses on how young people contribute to identifying and mitigating risks associated with research. For instance, one researcher observed that it is important to involve “*a Young Person’s Advisory Group where they can identify potential benefits and consequences of the research*” (R15). This involvement helps in understanding stakeholder impacts and managing risks more effectively (R2, R9, R15, R16). For instance, the involvement of young people throughout the project, the identification and management of “*risks and uncertainties but also making sure that the research aligns with young people’s needs*” (R12), which is “*almost more organic than ‘foreseeing’* [risks and unintended consequences]” (R8). In this sense, a young person pointed out that having “*young people with lived experience as part of advisory groups…is good to read around the risks of research but better to speak to young people directly*” (YP5) as it can aid the mitigation of potential risks by “*asking young people about possible unintended consequences and how*,* perhaps*,* to mitigate these consequences*” (R15), which is “*useful to understand stakeholder impact*,* and to consider risks and consequences before during and after project*” (R2).

Feature 5.4 (“*it can be triggering*”: *duty of care and protection*) addresses responsible considerations of involving young people in sensitive research topics in the field of youth mental health. Participants recognise that engaging young people in sensitive discussions around their mental health struggles can potentially be distressing. Some noted: “*these projects can often be very sensitive and involving young people with lived experience in discussions around self-harm and so on can be triggering*” (R12), “[there is the] *potential for unintentionally causing distress and harm is definitely there as we are tackling sensitive topics such as self-harm and suicide in digital youth*” (R17). Ensuring that young people’s well-being is safeguarded and providing adequate support are crucial aspects of responsible research practice. This approach is important because, as pointed out by a young person, “*there’s still a stigma around mental health and researchers should be mindful of this when creating spaces for young people to discuss issues related to their mental health*” (YP5). Therefore, it is important to monitor participants’ emotional states and provide support when needed: “[we need to be concerned about] *issues around enabling safe involvement*,* particularly where involvement is remote*” (R2), “[we should consider] *potential harms of participation and mitigating (e.g. by gatekeeping plus monitoring and sources of support)*” (R3), “[we have] *supportive staff members taken on responsibility for monitoring how young people are doing and debriefing and following up if needed*” (R17).

Feature 5.5 (“*we should not infringe adolescents’ privacy*”) highlights the importance of respecting young people’s privacy throughout the research process. Researchers stressed the need to address potential privacy risks and ensure responsible practices for research participants. For instance, one remarked: “[we should not infringe] *adolescents’ privacy from parents or other adults (including the researchers/businesses)*” (R18). This aligns with RRI principles that advocate for transparency and careful planning to protect participants’ privacy, which was also touched by researchers: “*we anticipate and address potential risks and impacts by carefully planning the research*,* particularly regarding participant privacy and ethical considerations*” (R9). Such practices are essential for maintaining responsible standards and respecting young people’s rights.

Feature 5.6 (“*it is deeply reflective*”: *the need for reflexivity throughout the process*) underscores the importance of reflexivity in implementing RRI practices. Researchers acknowledged that a reflexive approach is vital for identifying and addressing biases: “[it is important to] *being prepared to be reflexive about our own biases and sharing this with those you are working with or who are interested in your work*” (R21). Continuous reflections are also important to ensure ethical research practices: “[the RRI process requires] *continual self-reflective discussion within the team”* (R18) as they “*work really hard to try and understand the reasons or processes of implementing an intervention even prior to the development of it*” (R21) – these make RRI a “*deeply reflective* [process and it] *is helpful in uncovering and processing bias*” (R20). Therefore, continuous self-reflection and discussion within research teams are essential to maintain the integrity of the research process and ensure that it remains aligned with responsible practices (R2, R18, R20, R21).

In conclusion, this theme highlights the vital role of RRI principles in youth mental health research. They foster inclusive, participatory environments where young people’s input shapes research agendas. It also points to practical challenges, including managing limited resources, addressing sensitive topics, and protecting privacy.

### Theme 6: “*The two overlap hugely*”: RRI vs PPI

This theme explores the relationship between RRI and PPI, highlighting how both approaches prioritise stakeholder engagement, inclusivity, and the integration of lived experience. It also outlines their similarities and differences in the context of research.

Many participants noted that the RRI and PPI frameworks share substantial common ground. For example, one researcher argued that “*the two overlap[s] hugely. RRI is probably more freeform / less admin bound*” (R8), suggesting that while RRI and PPI both centre on involving stakeholders, RRI might offer more flexibility compared to the more structured approach of PPI. Another researcher echoed this sentiment, stating that both frameworks focus on “*centring the person in the middle of the development and ensuring their voices are heard throughout the research project*” (R21). This indicates that, for some participants, both RRI and PPI prioritise stakeholder engagement as a core component of their methodologies.

The relationship between RRI and PPI is also framed as one of inclusion and extension. Some researchers characterised PPI as a subset of RRI: “*PPI is a part of RRI*” (R2), “*PPI is a subset of RRI*” (R3), “*PPI is part of RRI where patients/public can be involved in RRI to identify intended and unintended benefits and consequences of the research*” (R15). This perspective aligns with the idea that while PPI specifically targets the involvement of patients and the public, whereas RRI encompasses a broader scope, integrating various stakeholders and addressing wider societal impacts. One researcher highlighted that “*RRI goes much further* [than PPI]. *It is an ongoing process that helps to consider the wider impacts of projects*” (R16), suggesting that RRI includes and extends beyond the principles of PPI to incorporate a more comprehensive view of research impacts.

Both approaches are seen as rooted in similar values and practices. For instance, it was noted that “*both approaches tend to include the voices of young people in the various cycles of the project*” (R5). This reflects a shared commitment to ensuring that stakeholder perspectives, particularly those of young people, are integral to the research process. Additionally, the necessity for meaningful involvement rather than mere tokenism is a common concern for both RRI and PPI. As one researcher articulated: “[both approaches are concerned with] *involving lived experience stakeholders in a meaningful way and not just as a tick box exercise*” (R12).

Despite these overlaps, the distinction between the two approaches is significant for some researchers. For instance, RRI is described as extending beyond the parameters of PPI such that “*PPI is part of RRI but RRI extends beyond simply involving a broader group of stakeholders*” (R11), suggesting that RRI encompasses a more expansive framework for integrating stakeholder feedback and addressing wider ethical and social concerns.

### YPAG’s reflections on the results

YPAG members acknowledged that the results reflected their first-hand experiences of their involvement in youth mental health projects. Table 4 showcases some of their key reflections and insights:

**Table 4:** YPAG’s key reflections on the results

Key Reflection	Supporting Excerpt
The integration of a Youth Co-chair was seen as crucial for challenging adult assumptions about youth mental health	- “[Youth Co-chair was] *really important because all of us*,* if not most of us*,* would not be here without them*” (Young Person 2) - “[this role] *has been a real strength that I’ve not seen* [in] *other projects like this*” (Young Person 1)
Communication and the use of jargon were highlighted as key issues that should be anticipated and addressed from the outset	- “*So one of the things that comes up when you mention communication gaps is like jargon*” (Young Person 3) - “*There was one time I was in a meeting and there was a word that was used. I think it was just the word “range” but it’s like an everyday word that had a second meaning*” (Young Person 4) *- “*[the programme’s huddles - periodic in-person meetings] *have been really good with breaking down some of those barriers in communication*” (Young Person 3)
It is important to have a holistic and comprehensive view of the lack and/or quality of involvement	- “[This is] *especially with young people with mental health*,* it can be hard to find the time and if you have not only the time*,* but if you have mental health challenges that can get in the way*,* you know… There was some real significant reasons why I couldn’t be involved*” (Young Person 4) - [The lack of reciprocal communication can reduce motivation as] *sometimes*,* when you’re kind of not communicated back with* [by researchers] *you kind of just like*,* ‘OK*,* what’s the point?’ kind of thing. Whereas obviously if you know that as much as you care about the project people care about you too*,* you want to give more to it*” (Young Person 5)
It is important to constantly challenge the ‘kids’ and ‘grown-up’ tables culture	- “[reflexive sessions such as this one are] *really enlightening and really*,* really good for*,* I think us as young people*,* you know*,* but also researchers as well to kind of hear it from different perspectives and those who were like in it day-to-day with the lived experience*” (Young Person 1) - “[The] *relationship building* [between young people and researchers] *is really*,* really important* [to overcome such a culture]” (Young Person 1)
The programme has a range of identities and backgrounds regarding inclusivity and diversity – but there is always room for improvement	- “*as you can see from here*,* there is diversity in so many different forms*,* people with different diversity in terms of backgrounds*,* ages*,* what people do*,* they’re different lived experiences*,* like absolutely everything*” (Young Person 6) - [Diversity is not inherently difficult to achieve], *it’s all about how hard you try to be diverse*,* if that makes sense. like the famous quote ‘no one’s hard to reach. It’s how hard you try to reach them’. I feel like that’s been my quote of the project*” (Young Person 2)
Empathy is key as youth mental health projects can be triggering for young people	- *“*[It] *is a very real subject for everyone involved*,* either* [from a] *young person’s perspective or* [that of] *researchers… but I think it’s easy to forget that you’re dealing with humans*” (Young Person 2) - “[As a researcher] *you’re also dealing with sensitive topics and it’s like*,* how do we take a more empathetic approach when dealing with these things*,* but still achieving those objectives that we have?*” (Young Person 7)
Young people tend to be generally comfortable with data sharing and privacy issues, as long as anonymity and autonomy are respected	- “[As long as] *you have a choice of how your data is used*,* like if it’s anonymous*,* if your first name*,* no name*,* full name. So that’s OK*” (Young Person 2) - “*allowing choices to disclose some information*,* I think also continually check it in*,* in and asking questions is a great way to progress in this space*” (Young Person 3) “*it’s also about trusting*,* feeling comfortable to share that information and knowing that it’s safe and that you have choice and autonomy*” (Young Person 4)

Note: for a more detailed and comprehensive discussion of these accounts, see Mendes et al. [51]

## Discussion

As shown in Table 3, the six themes can be distributed across the KAP dimensions. Theme 1 falls within the ‘Knowledge’ dimension, as it captures participants’ understandings of youth mental health and reflects its broad conceptualisation. Analytically, positioning Theme 1 within ‘Knowledge’ is also consistent with an RRI-oriented approach, since these understandings and conceptions provide the context for applying responsible principles: anticipatory and reflexive practice requires a shared, explicit conceptualisation of the core construct (in this case, ‘youth mental health’) before examining subsequent attitudes and practices, such as co-production and engagement [2, 4, 10]. Within an RRI framework, context is not peripheral but foundational, as it shapes the values, expectations, and lived realities that research must anticipate and address [10, 57, 58]. This emphasis echoes RRI values regarding anticipation, reflexivity, engagement, and action, which stress the need to identify potential risks, reflect critically on assumptions, and engage inclusively with those most affected [2, 9, 10]. In practice, these conceptual groundings have implications for research design and policy: without agreement on what constitutes ‘youth mental health’, subsequent interventions risk being misaligned with the lived realities they intend to serve [7, 13].

Building on this foundation, young people especially emphasised how social exclusion, economic precarity, and digital harm affect mental health, reinforcing RRI’s imperative to ground research in lived experience rather than abstract assumptions [4]. Their nuanced insights underscored the value of co-producing definitions and metrics [23], aligning with RRI’s procedural emphasis on transparency, deliberation, and mutual responsiveness [11]. Moreover, conceptualising youth mental health as a continuum, ranging from flourishing to languishing, and shaped by both personal (e.g., physical and psycho-emotional attributes) and interpersonal (e.g., social relationships and cultural contexts) determinants resonates with established frameworks in the field [59, 60]. This perspective highlights the importance of integrating RRI principles into youth mental health initiatives: aligning research practices with such complex realities can support the co-creation of interventions that are both contextually relevant and ethically sound [7, 13, 61]. Whilst young people highlighted issues such as digital harms as key factors, researchers focused more on biopsychosocial models linking individual and systemic issues. These complementary perspectives underscore the need for anticipatory and inclusive practices that not only acknowledge but also integrate these differences, offering more robust and equitable pathways for policy and intervention [62–64].

Theme 2 aligns most closely with the ‘Attitudes’ and ‘Practices’ dimensions, as it foregrounds researchers’ attitudes and behaviours towards valuing young people’s perspectives and a willingness to have their assumptions challenged (Features 2.1 and 2.2). In the context of youth mental health, this focus holds particular relevance, as research seldom places young people’s own reflections at the forefront, even with growing backing from institutions and funders, focusing instead on implementation processes or researchers’ perspectives [65–67]. Theme 2 also evidences practices like involving young people to ‘get the design right’, co-developing methods, interpreting data together, informing policy, and embedding youth input in decision-making and outcome design (Feature 2.3) – it is possible to notice a minor knowledge element in Feature 2.3 (e.g., recognising risks of exclusion leading to unwanted/unused technologies and/or tools and interventions), but this functions mainly as justificatory context for the attitudes and practices. Accordingly, Theme 2 overall demonstrates how pro-youth values are translated into collaborative RRI practices.

Including stakeholder perspectives is central to RRI [2], and Theme 2 illustrates how young people help to reframe priorities and enhance the clarity and relevance of research tools – a good example, within the programme researched, is the development process of CaTS-APP, designed to support young people who self-harm by helping users and professionals (such as therapists) to understand the thoughts, feelings, and events leading to self-harm episodes [7]. Young people’s input reflects RRI’s generative potential for creativity and innovation [3]. However, this potential depends on intentional participatory structures, such as the Youth Co-chair and regular huddles, which exemplify the procedural infrastructures needed to ensure inclusive and equitable research [4, 12] – our YPAG has endorsed this view, considering initiatives like the Youth Co-chair as essential for challenging adult assumptions about youth mental health and for enabling meaningful youth involvement.

Theme 3 encompasses the ‘Practices’ and ‘Attitudes’ dimensions. Its primary emphasis lies in concrete collaborative practices that strengthen research, such as consulting young people, embedding their feedback into the design of research tools and interventions, and recognising that disagreements can foster conclusions that extend ‘outside the box’ (Feature 3.1). These practices align with expectations in the field that young people should be meaningfully involved throughout the research and development lifecycle [13, 68, 69]. However, responsible approaches are essential to prevent exploitative or tokenistic practices [64–67]. This is where the attitudinal norms highlighted hereby (Feature 3.2) become particularly relevant, as they underscore the importance of clarity, honesty, open communication, mutual respect, understanding, and compromise, which was also emphasised by our YPAG – signposting what Mendes et al. [51] have described as the ‘dialogic and caring dimensions’ of co-production with young people in the mental health field. A smaller ‘Knowledge’ element is also present in Theme 3, where young people in particular articulated distinctive expertise that enhances researchers’ understandings of youth mental health (Feature 3.1). Yet this primarily serves as a justificatory context for the attitudinal commitments and co-production practices that operationalise responsible involvement.

Theme 4 aligns primarily with the ‘Attitudes’ and ‘Practices’ dimensions. Attitudinal challenges emerge around communication, equity, and mutual recognition. For example, researchers acknowledge difficulties in making academic language accessible and ensuring consistent involvement, while young people emphasise the persistence of hierarchical dynamics, described as a ‘kids’ and ‘grown-up tables’ culture (Features 4.1 and 4.3). These hierarchies resonate with analyses of epistemic injustice, in which children and young people are routinely treated as less reliable or important sources of knowledge and are not always provided with the interpretive resources needed to make sense of their experiences [27, 28, 30]. Also, these examples reflect underlying beliefs and orientations towards partnership, underscoring the need to foster openness, respect, and inclusivity. From this perspective, characterising young people’s intermittent or constrained participation simply as ‘flakiness’ risks reinforcing adult-centric expectations of reliability and availability, and may obscure how institutional timetables, communication styles, and limited flexibility on the part of adults contribute to patterns of uneven engagement [70–73]. This dynamic is exemplified in R3’s description of needing involvement to be “subjectively worthwhile for them [i.e., the young person]”, which can, mistakenly position the researcher’s judgement about what counts as ‘worthwhile’, rather than inviting young people to define value and meaning on their own terms. Work on epistemic injustice in mental health research cautions that such interpretative privilege can reproduce hierarchies in which professional or academic standpoints are treated as more authoritative than lived experience perspectives [74, 75].

Adopting an RRI lens, therefore, requires researchers to practise reflexivity about their positionality and to foreground youth-defined accounts of what makes involvement feasible, helpful, or burdensome, rather than assuming that adult interpretations should take precedence [7, 8, 51, 75]. In this regard, our YPAG highlighted the importance of taking a more nuanced view of what may appear as a ‘lack of interest’ or ‘inconsistent involvement’. They suggested that such issues might be linked to the sensitivity of the topics under discussion and/or young people’s current mental health struggles, which may limit or hinder further participation. This aligns with recent work calling for ‘radical listening’ to young people’s accounts of their own constraints and priorities, so that adult partners do not inadvertently reproduce epistemic injustices by privileging institutional norms over youth-defined realities [70, 74, 75]. Such reflexive attention can help prevent miscommunication or misinterpretation dynamics that hinder collaboration and compromise researchers’ duty of care, while also guarding against subtle forms of epistemic injustice in which youth constraints are pathologised rather than understood in context [74, 75]. This perspective is important to avoid miscommunication or misinterpretation dynamics that can hinder co-production as well as compromise researchers’ duty of care, particularly when ‘flakiness’ is perceived, where, in fact, there may be indications of young people’s involvement and/or mental health struggles.

Structural issues like communication gaps, uneven engagement, and power imbalances mirror concerns widely discussed in the youth mental health literature [13, 20, 23, 39, 51, 53, 62, 63, 76–82]. These issues also relate to epistemic justice as they highlight how language, terminology, and representational practices can entrench or disrupt such power asymmetries, with children and other marginalised groups often positioned as objects rather than sources of knowledge [29, 51, 83–86]. RRI principles can address such challenges [8, 11, 51], particularly because of their emphasis on care and trust-building [87, 88], which includes recognising and resourcing the emotional labour, time pressures, and vulnerabilities young people face when contributing to mental health initiatives – for further discussion regarding tensions inherent in researcher-youth partnerships, including power dynamics and risks of tokenism, please see Mendes et al. [8, 51].

Theme 4 also strongly evidences practices associated with sustaining collaboration, such as simplifying jargon, maintaining consistent engagement despite competing demands, and adapting strategies to manage resource-intensive processes (Features 4.1, 4.2 and 4.4). These practices highlight the behavioural efforts required to operationalise responsible involvement in contexts where participation is complex and demanding [7, 39, 51, 53]. In addition, our YPAG emphasised that communication and jargon issues must be anticipated and actively mitigated in collaborations between young people and researchers in the youth mental health field. While minor ‘Knowledge’ elements appear in this theme (such as recognising that wide consultation can be “very resource demanding” and identifying risks of disengagement) these primarily serve as contextual justification for the attitudinal and practical efforts needed to bridge differences in expertise and sustain equitable adult–youth partnerships.

Theme 5 maps across the ‘Attitudes’, ‘Practices’, and ‘Knowledge’ dimensions, with a clear predominance of practices. Concrete practices are most evident in the ways RRI structures inclusivity, participatory engagement, risk identification, safeguarding, privacy protection, and reflexivity throughout the research process (Features 5.1–5.6). These demonstrate how RRI can be operationalised in youth mental health research, from consulting diverse young people to embedding reflexive processes that sustain responsible standards [7, 8, 51]. Viewed through an epistemic justice lens, such RRI-informed participatory structures can be read as practical routes to redistributing whose knowledge counts in shaping research questions, methods, and decisions, thereby challenging adult-centric epistemic hierarchies [7, 23, 39, 51, 53, 83, 84, 89]. Attitudinal commitments also underpin these practices, particularly in valuing authentic youth involvement, recognising the duty of care when addressing sensitive topics, and respecting young people’s privacy (Features 5.2, 5.4, and 5.5) – concerns that the RRI literature addresses under risk anticipation and ethical acceptability [1, 12]. However, our analysis also indicates that safeguarding cannot be reduced to risk avoidance alone: overprotective or paternalistic responses risk marginalising young people’s own priorities and decision-making capacities, even when motivated by care [8, 51, 90]. The emphasis on autonomy and privacy further resonates with RRI’s call for transparency and agency across the research/innovation lifecycle [5, 11]. This is consistent with qualitative work in youth mental healthcare showing that relationally attuned care, in which young people feel listened to, respected, and able to influence aspects of their support, is central to their sense of safety, trust, and engagement with services [51, 90]. These commitments align with RRI’s focus on responsiveness and safeguarding, grounding research and innovation in the lived experiences of stakeholders – a consideration that is especially critical in emotionally nuanced areas such as youth mental health [7, 8, 10, 18–20, 51, 73, 76, 82, 90–93].

A minor knowledge element is present in Theme 5 as both researchers and young people articulate RRI’s contribution to identifying risks, benefits, and unintended consequences (Feature 5.3), as well as in framing reflexivity as a means of uncovering bias (Feature 5.6). However, these function primarily as justificatory context that reinforces why the attitudinal and practical dimensions of RRI are critical for ensuring inclusivity, safeguarding, and meaningful youth engagement – key pillars of RRI [1, 2, 4].

Theme 6 sits primarily within the ‘Knowledge’ and ‘Attitudes’ dimensions. The knowledge element is evident in participants’ articulation of conceptual overlaps and distinctions between RRI and PPI, including the recognition that both approaches prioritise inclusivity, lived experience, and stakeholder engagement, while RRI extends further to encompass wider societal impacts (e.g., framing PPI as a subset of RRI) – this resonates the literature in this area, which positions PPI with a focus on specific research stages, while RRI takes a continuous, value-driven approach with integrated governance [2, 12]. Attitudinal aspects in Theme 6 underpin this comparative framing, reflecting researchers’ values around meaningful rather than tokenistic involvement, centring young people’s voices, and acknowledging the ethical importance of participation. Practices are less central here but remain implicit in the ways participants describe operationalising both approaches, such as embedding stakeholder voices across project cycles. Overall, this theme demonstrates how participants situate RRI and PPI as complementary yet distinct, with knowledge of their conceptual relationship functioning as the basis for attitudinal commitments towards responsible and inclusive research.

### Implications for involvement practice in youth mental health

Taken together, the six themes illustrate how RRI principles can be translated into concrete practices that support youth involvement in mental health research. Below, we draw on these results to outline key implications for those designing, conducting and governing youth mental health projects, with the aim of strengthening both the quality and impact of involvement:

1. ***work towards shared understandings of ‘youth mental health’ and their implications for the involvement process***: involvement practice should start from shared, explicit understandings of youth mental health as a continuum shaped by physical, psychological, social and economic determinants, co-articulated with young people rather than assumed in advance. This helps to ensure that subsequent attitudes, methods and interventions are aligned with young people’s lived realities and priorities;
2. ***establish and resource structured roles for young people***: formal roles such as Youth Co-Chairs, advisory groups and regular “huddles” illustrate how young people can be integrated and influence priorities, methods and decisions across project stages. These structures require time and resources, but they are central to sustaining involvement that moves beyond one-off consultation and supports young people to shape research agendas in ways they find meaningful;
3. ***recognise young people as epistemic partners with distinctive expertise***: young people’s accounts show that they offer expertise and experience and help researchers to increase understanding of how they conceptualise young people’s mental health, often generating conclusions that neither group would reach alone. In practice, this calls for treating lived and experiential knowledge as a core source of insight in the design of tools, the interpretation of data and decisions about impact;
4. ***anticipate and address communication barriers as part of involvement design***: difficulties in keeping up with jargon and translating academic terms into accessible language are recurrent challenges. Involvement practice should therefore build in time and strategies to simplify language, check understanding, create spaces where questions about terminology are welcomed, and use formats (such as huddles) that help to break down barriers related to hierarchies and language;
5. ***adopt a nuanced***,*** dialogic view of “inconsistent” engagement***: experiences that adults label as “flakiness” co-exist with young people’s accounts of mental health struggles, time pressures and a lack of reciprocal communication. Rather than framing intermittent involvement as a deficit on the part of young people, involvement practices should attend to the emotional weight of youth mental health topics, build in flexibility and treat patterns of engagement as prompts for relational checking in rather than grounds for judgement;
6. ***actively challenge ‘kids’ and ‘grown-up table’ dynamics***: young people’s references to a “kids table” versus a “grown-up table” indicate persistent hierarchies within involvement spaces. Relationship-building, shared reflexive sessions and explicit efforts to centre young people’s perspectives are needed to disrupt such cultures and support more equitable adult-youth partnerships in which young people feel “really valued” and are able to influence outcomes;
7. ***take advantage of RRI principles to hold together inclusivity***,*** safeguarding and reflexivity***: participants’ descriptions of inclusivity, diversity, risk identification, duty of care, privacy protection and “deeply reflective” practice show that RRI offers a practical framework for involvement work. In practice, this means designing processes that invite diverse young people into projects, enable them to identify benefits and unintended consequences, attend carefully to potential “triggering” topics and sustain ongoing reflexive discussion within teams about how responsibilities are being enacted;
8. ***protect privacy while supporting choice and autonomy in data sharing***: respecting adolescents’ privacy from parents or other adults and planning carefully around data use are central to responsible involvement. Young people’s reflections on anonymity, options for how their data are used and the importance of trusting and feeling comfortable suggest that involvement practice should foreground clear choices about data, regular check-ins and reassurance that autonomy will be respected;
9. ***be explicit about how RRI and PPI are being used in practice***: participants’ descriptions of PPI as part of, or a subset of, RRI, and their emphasis on meaningful rather than tokenistic involvement, imply a practical need to state which framework is being used, for what purposes, at which stages and whether there are any complementary roles between PPI and RRI. Making these choices explicit can guide how stakeholder voices are embedded across project cycles and help to ensure that the broader, anticipatory aims of RRI are not reduced to procedural PPI requirements;
10. *** acknowledge the labour and constraints of involvement in reporting and planning***: accounts of involvement as “very resource demanding”, difficulties finding a suitable time to engage young people and the emotional strain of sensitive topics point to the need to acknowledge this labour explicitly. Involvement practice should therefore build in realistic time, support and resourcing for both young people and researchers, and future reporting should describe these efforts and constraints so that expectations about youth involvement are grounded in the realities of what meaningful collaboration entails.

### Strengths and limitations

This study’s novelty lies in its empirical focus on how RRI principles are interpreted and enacted within a real-world youth mental health programme. By incorporating the perspectives of both researchers and youth collaborators, it provides concrete, practice-based insights into the ethical, procedural, and relational dimensions of RRI in this field. A further strength is the comparative lens afforded by examining the perspectives of young people alongside those of researchers. The active involvement of our YPAG throughout interpretation and dissemination further exemplifies how inclusion, reflexivity, and responsiveness can be operationalised in practice. Methodologically, combining Reflexive Thematic Analysis with the Knowledge, Attitudes, and Practices framework enabled a structured yet flexible approach to situating participants’ accounts within an RRI lens. Together, these elements contribute to advancing the emerging discourse on responsible youth involvement and provide new empirical evidence to guide future adaptations of RRI in psychosocial research contexts.

Regarding limitations, although the online survey facilitated broad participation across the programme’s work packages, the dataset was limited to individuals directly involved in the programme, which may reduce its transferability to other contexts. Reliance on self-report also introduces risks of social desirability and selective recall – issues we sought to address responsibly by involving our YPAG to review and critique the results. Despite these limitations, this study offers important empirical evidence of how RRI principles can be enacted in practice and highlights infrastructures (such as youth co-chairs, communication support, and flexible funding) required to sustain meaningful and equitable involvement in adolescent mental health research.

## Conclusions

This study did not aim to present RRI as a comprehensive or fixed model. Rather, it explored its potential and transformative applications in the youth mental health field, so the results and discussion should be read as signposting plausible pathways for incorporating RRI into youth-centred projects, rather than as a definitive blueprint.

Overall, the analysis underscores RRI’s relevance to youth mental health research when equity, reflexivity, anticipation, and responsiveness are deliberately integrated into project design and delivery. In this context, RRI is best understood as a dynamic practice that is co-shaped through relationships and decision-making between researchers and young people, with implications for both ethical integrity and social impact. Young people’s involvement can challenge researchers’ assumptions and strengthen the development of interventions and tools by grounding choices in lived realities, but this value is contingent on interpersonal foundations such as mutual respect, open communication, and genuine recognition, without which collaboration can drift towards tokenism.

Meaningful youth involvement in mental health research requires early engagement, clear influence pathways, and supported participatory roles. Structured support for intergenerational dialogue and flexible, well-resourced processes are also essential. If funders and institutions seek sustained equity and inclusion, they should explicitly resource the relational and iterative work required for RRI-aligned practice, including safeguarding-sensitive forms of participation that protect young people without displacing their agency. In this way, RRI can complement more established approaches (e.g., PPI) by providing a broader anticipatory and systemic lens that helps move youth mental health research beyond one-off consultation and towards more inclusive, caring, and accountable co-production.

## Supplementary Information

Below is the link to the electronic supplementary material.

Supplementary Material 1: Additional File 1 – Survey Questions

Supplementary Material 2: Additional File 2 - First level of analysis (open coding)

Supplementary Material 3: Additional File 3 - Second Level of Analysis: generating initial themes

Supplementary Material 4: Additional File 4 - Reflexivity

Supplementary Material 5: Additional File 5 - GRIPP2 short form

## Data Availability

Given the sensitive nature of the discussions and participant preferences, full transcripts are not available; however, anonymised excerpts and additional files are provided to ensure transparency.

## Footnotes

1 Consistent with our previous work [8, 51], in this paper, we use ‘youth mental health’ to refer to the emotional, cognitive, behavioural and relational well-being of young people aged approximately 10–24, a developmental span that extends beyond narrower definitions of adolescence and encompasses transitions through puberty, secondary schooling, and emerging adulthood. This period is marked by rapid biological, psychological, and social change, a high incidence of onset for most mental disorders, and shifting social roles and service contexts, which together distinguish ‘youth mental health’ conceptually and practically from child-only or adolescent-only research [94, 95].

2 Within the Digital Youth Programme, ‘Co-chair’ is a formal role for young people who share leadership with adult researchers. They help set agendas, facilitate meetings, represent youth perspectives, and ensure that discussions and decisions reflect intergenerational equity. By doing so, Co-chairs move beyond tokenism, redistribute power, and position young people as genuine partners and leaders in adolescent mental health and digital innovation.

3 We acknowledge Braun and Clarke’s [46] critique that themes and subthemes can often be presented as functional topic summaries rather than interpretative narratives. However, we align with Mendes et al. [49], who emphasise the importance of making qualitative findings accessible to policymakers and decision-makers, who tend to engage with practical, descriptive outputs [47]. To balance interpretative depth with accessibility, we used in vivo coding for naming themes and features while exploring deeper meanings within each feature.

4 Drawing on our previous work on PPI/RRI in youth mental health and on coproduction with young people [7, 8, 51], we use “meaningful participation” to refer to forms of youth involvement that go beyond one-off consultation. Such participation can be characterised by early and, ideally, sustained engagement across project stages, clear opportunities to influence agendas and decisions, reciprocal recognition of lived and experiential expertise, appropriate support and safeguarding (including attention to emotional labour and accessibility), and a genuine capacity for young people’s contributions to shape both processes and outputs, rather than being merely tokenistic.

## Abbreviations

AREA: Anticipate, Reflect, Engage, Act
PPI: Patient and Public Involvement
RRI: Responsible Research and Innovation
UK: United Kingdom
UKRI: United Kingdom Research and Innovation
YP: Young People
YPAG: Young People Advisory Group
