# Supplementary material for: Exploring Responsible Research and Innovation (RRI) in youth mental health: reflections from researchers and young people
Source: Res Involv Engagem. 2026 Feb 6;12:31. doi: 10.1186/s40900-026-00848-x (PMC12973806; doi:10.1186/s40900-026-00848-x)
Supplement: Supplementary file 1 — Supplementary Material 1: Additional File 1 – Survey Questions [file 40900_2026_848_MOESM1_ESM.pdf]

Additional File 1 – Survey Questions

Article: Exploring Responsible Research and Innovation (RRI) in Adolescent Mental Health: Reflections from Researchers and Young People

Journal: Research Involvement and Engagement

Authors: Josimar Antônio de Alcântara Mendes; Mathijs Lucassen; Sarah Doherty; Ayan Mahamud; Carolyn Ten Holter; Chris Greenhalgh; Ellen Townsend; Marina Jirotka

Survey Open-ended Questions

1) How would you define 'mental health'?
2) In your opinion, what are the main determinants of adolescent mental health?
3) How would you describe your work with young people within the programme? [Researchers]
4) What do you understand by 'Responsible Research and Innovation'? [Researchers]
5) Regardless of your understanding or previous experiences with RRI, what do you think 'Responsible Research and innovation' (RRI) refers to? [Young People]
6) Based on your knowledge on 'Responsible Research and Innovation' (RRI), how do you think an RRI approach could contribute to collaborative projects involving young people? [Researchers]
7) How do you think an RRI approach can be useful for projects focused on adolescent mental health?
8) What would you consider a risk and/or unintended impact/consequence under a responsible approach to adolescent mental health? [Researchers]
9) What risks and/or unintended impacts/consequences do you think researchers should consider when they are addressing adolescent mental health? [Young People]
10) Considering your work within your work package, do you foresee risks and unintended impacts/consequences? If yes, how do you foresee them? [Researchers]

11) How do you think researchers could foresee and mitigate risks and/or unintended impacts/consequences when they are addressing adolescent mental health? [Young People]
12) Considering your work within your work package, do you mitigate risks and unintended impacts/consequences? If yes, how do you mitigate them? [Researchers]
13) In your opinion, what are the main advantages of collaborating with young people in this project? [Researchers]
14) In your opinion, what are the main advantages of collaborating with researchers in this project? [Young People]
15) In your opinion, what are the main challenges of collaborating with young people in this project? [Researchers]
16) In your opinion, what are the main challenges of collaborating with researchers in this project? [Young People]
17) What do you think is important whenever researchers and adults are collaborating with young people? [Young People]
18) How do you think a responsible approach could address diversity?
19) How do you think a responsible approach could address biases?
20) Do you see any overlaps between RRI and 'Patient and Public Involvement' (PPI)? If yes, what they might be? [Researchers]
