# Supplementary material for: Exploring Responsible Research and Innovation (RRI) in youth mental health: reflections from researchers and young people
Source: Res Involv Engagem. 2026 Feb 6;12:31. doi: 10.1186/s40900-026-00848-x (PMC12973806; doi:10.1186/s40900-026-00848-x)
Supplement: Supplementary file 2 — Supplementary Material 2: Additional File 2 - First level of analysis (open coding) [file 40900_2026_848_MOESM2_ESM.pdf]

Additional File 2

Article: Exploring Responsible Research and Innovation (RRI) in Adolescent Mental Health: Reflections from Researchers and Young People

Journal: Research Involvement and Engagement

Authors: Josimar Antônio de Alcântara Mendes; Mathijs Lucassen; Sarah Doherty; Ayan Mahamud; Carolyn Ten Holter; Chris Greenhalgh; Ellen Townsend; Marina Jirotka

First level of analysis (open coding)

Name	Description	Sources	References
**RRI Conceptions**	Ideas, definitions, and general conceptions surrounding the Responsible Research and Innovation (RRI) approach	29	31
**Mental Health Definition**	Definitions and perspectives on the concept of mental health	28	36
**RRI & Young People**	Ideas, conceptions, and practices that illustrate how the RRI approach can be applied in collaborative projects involving young people	26	49
**Determinants of Young People Mental Health**	Recognitions of factors that influence young people's mental health	24	30
**Advantages of Collaborating with Young People**	Recognitions of the advantages and benefits of collaborating with young people in projects related to youth mental health	23	33
**Foreseeing Potential Risks**	Recognitions of potential risks and negative impacts that may arise when addressing youth mental health in collaborative projects	22	48
**Challenges of Collaborating with Young People**	Recognitions of the challenges encountered when collaborating with young people in projects within the field of youth mental health	21	32
**RRI and Diversity**	Recognitions of how the RRI approach can address diversity-related issues	21	22
**RRI & Biases**	Recognitions of how the RRI approach can address and mitigate biases	18	20
**Mitigating Potential Risks**	Actions and practices that can help mitigate potential risks and negative impacts in collaborative projects focused on youth mental health	16	26

Name	Description	Sources	References
**Possible Overlaps between RRI and PPI**	Recognitions of potential overlaps between Responsible Research and Innovation (RRI) and Patient and Public Involvement (PPI)	14	17
**Interactions with Young People**	Reflections, impressions, and considerations regarding the process of interacting with young people	8	13
