# Supplementary material for: Exploring Responsible Research and Innovation (RRI) in youth mental health: reflections from researchers and young people
Source: Res Involv Engagem. 2026 Feb 6;12:31. doi: 10.1186/s40900-026-00848-x (PMC12973806; doi:10.1186/s40900-026-00848-x)
Supplement: Supplementary file 3 — Supplementary Material 3: Additional File 3 - Second Level of Analysis: generating initial themes [file 40900_2026_848_MOESM3_ESM.pdf]

Additional File 3

Article: Exploring Responsible Research and Innovation (RRI) in Adolescent Mental Health: Reflections from Researchers and Young People

Journal: Research Involvement and Engagement

Authors: Josimar Antônio de Alcântara Mendes; Mathijs Lucassen; Sarah Doherty; Ayan Mahamud; Carolyn Ten Holter; Chris Greenhalgh; Ellen Townsend; Marina Jirotka

Second Level of Analysis: generating initial themes

Number of candidate themes: 9

Number of features: 23

Candidate Theme 1: "*Is the project valuable to them?*": researchers' keenness to collaborate with young people

General description: This theme captures researchers' recognition of the importance of young people's perspectives in youth mental health projects. It reflects their openness to being challenged on assumptions, the value they place on empowering young people, and their belief in young people's role in ensuring responsible and ethical approaches. Ultimately, it underscores researchers' commitment to making projects relevant and valuable to the young people involved.

Feature (a): Being challenged on my own assumptions and focusing on what is important for them

Description: This feature highlights researchers' openness to questioning their assumptions and prioritising young people's needs and perspectives in their work.

Source: [R1, R2, R8, R14, R17, R18, R21]

Feature (b): Acknowledging young people's perspective

Description: This feature underscores the importance of recognising and incorporating young people's viewpoints to ensure the relevance of youth mental health projects.

Source: [R2, R3, R5, R9, R12, R13]

Feature (c): Young people helping to ensure responsible approaches

Description: This feature reflects how young people contribute to responsible research practices in youth mental health projects.

Source: [R2, R3, R4, R5, R7, R15, R16, R17, R18, R20, R21]

Feature (d): Empowering young people

Description: This feature focuses on the importance of giving young people the agency to influence and shape research, ensuring meaningful collaboration.

Source: [R18]

Candidate Theme 2: "*We offer expertise and experience they may not have*": young people's perspectives on working with researchers

General description: This theme captures young people's perspectives on their roles in youth mental health projects. It highlights their contributions to crucial research, providing expertise that researchers may lack. Additionally, it reflects how participation inspires young people and addresses their needs in partnerships with adults, emphasising support, respect, and meaningful collaboration.

Feature (a): Aiding crucial research

Description: This feature highlights how young people offer valuable insights and expertise that enhance the quality and relevance of research in youth mental health projects.

Source: [YP1, YP2, YP4]

Feature (b): Inspiration

Description: This feature reflects how participation in these projects inspires young people, fostering motivation and a deeper connection to the work being done.

Source: [YP1]

Feature (c): Needs in the relationship with adults

Description: This feature emphasises the importance of addressing young people's needs in their interactions with adults, focusing on support, respect, and a balanced, collaborative dynamic.

Source: [YP1, YP2, YP4, YP5]

Candidate Theme 3: "*It is hard to bridge different types of expertise*": navigating complexities in Adult-Youth partnerships

General description: This theme explores the complexities of Adult-Youth partnerships in youth mental health projects, focusing on the challenges both researchers and young people face. It addresses difficulties in communication, ensuring young people's true involvement, and maintaining equity within these partnerships. Additionally, the theme highlights the struggle to promote genuine transformation, particularly within mental health institutions.

Feature (a): Bridging the communication

Description: This feature highlights the challenges in establishing effective communication between researchers and young people, emphasising the need for strategies to overcome misunderstandings and ensure clear, reciprocal dialogue.

Source: [R5, R12, R15, R16, R17, R18, R20, R21, YP2, YP4]

Feature (b): Bridging the involvement

Description: This feature addresses the difficulties in securing genuine participation from young people in partnerships, focusing on ensuring that their involvement is meaningful and impactful within youth mental health projects.

Source: [R2, R3, R4, R9, R13, R15]

Feature (c): Bridging the equity: challenging the 'kids' and 'grown up' tables culture

Description: This feature examines the struggle to ensure equity in partnerships by challenging the traditional power dynamics and hierarchical structures that often marginalise young people, aiming to foster a more balanced and inclusive approach.

Source: [R17, YP2, YP5]

Feature (d): Bridging the transformation

Description: This feature explores the challenges in promoting true transformation within partnerships, particularly within mental health institutions, highlighting the need to integrate diverse perspectives to achieve significant and lasting change.

Source: [R19, R20]

Candidate Theme 4: "*A series of emotional, cognitive, behavioural, and physical spectrums ranging from healthy to unhealthy*": Definitions and Determinants for 'Youth Mental Health'

General description: This theme explores how researchers and young people define and understand youth mental health. It includes perceptions of physical issues, cognitive, psychological, and emotional traits, as well as family, social, and economic factors. This theme highlights the diverse influences that shape the definition and determinants of mental health in young people, encompassing a range of healthy and unhealthy states.

Feature (a): Physical traits

Description: This feature highlights perceptions of how physical issues and health conditions are related to the definition and determinants of youth mental health, examining their impact on overall mental well-being.

Source: [R1, R4, R11, R12, R15, R17, YP1, YP2]

Feature (b): Cognitive, psychological or emotional traits

Description: This feature addresses perceptions of cognitive, psychological, and emotional factors that influence mental health, focusing on how these traits shape definitions and determinants of mental health in young people.

Source: [R1, R3, R5, R6, R7, R9, R10, R11, R12, R13, R15, R16, R17, R20, YP1, YP2, YP3, YP4, YP5]

Feature (c): Family, social and economic traits

Description: This feature explores the role of family, social, and economic factors in defining and determining youth mental health, emphasising how these contextual elements affect mental well-being and contribute to understanding mental health issues.

Source: [R1, R2, R4, R5, R6, R8, R9, R10, R11, R15, R17, R20, YP1, YP2, YP4, YP5]

Candidate Theme 5: RRI conceptions

General description: This theme explores the conceptions of Responsible Research and Innovation (RRI) as understood by researchers. It includes principles such as conducting research ethically for the common good, addressing biases, incorporating diverse perspectives, and ensuring transparency in the research process. It reflects a commitment to ethical and inclusive research practices.

Source: [R2, R13, R14, R15]

Candidate Theme 6: Foreseeing and Mitigating Potential Risks and Unintended Impacts

General description: This theme addresses the need to foresee and mitigate potential risks and unintended impacts in youth mental health projects. It includes perceptions of the importance of youth participation, researchers' duty of care, privacy concerns, and the utility of interventions. It also highlights the need to ensure diversity and maintain reflexivity throughout the project.

Feature (a): Ensuring participation

Description: This feature highlights the importance of effectively involving young people in youth mental health projects to ensure their voices are heard and their contributions are valued.

Source: [R2, R4, R8, R11, R12, R13, R18, YP1, YP2, YP5]

Feature (b): Duty of care & Protection

Description: This feature emphasises researchers' responsibility to safeguard young people involved in projects, ensuring their well-being and protection throughout the research process.

Source: [R2, R3, R4, R8, R11, R12, R13, R16, R17, YP5]

Feature (c): Privacy concerns

Description: This feature addresses the need to protect the privacy of participants by managing data confidentiality and addressing any concerns related to the handling of personal information.

Source: [R9, R18]

Feature (d): Ensuring diversity

Description: This feature underscores the need to promote diversity within youth mental health projects, ensuring that a range of perspectives and experiences are represented and considered.

Source: [YP1, YP4]

Feature (f): Reflexivity

Description: This feature highlights the importance of maintaining reflexivity throughout youth mental health projects, encouraging ongoing reflection on practices and their impacts to ensure responsible and effective outcomes.

Source: [R2, R3, R4, R5, R18, R20, R21]

Candidate Theme 7: RRI Caveats

General description: This theme addresses the caveats of Responsible Research and Innovation (RRI), highlighting challenges such as the potential for extensive consultations to become time-consuming and costly. It reflects on the practical limitations and trade-offs associated with adhering to RRI principles.

Source: [R8]

Candidate Theme 8: RRI vs PPI

General description: This theme explores the relationship between Responsible Research and Innovation (RRI) and Patient and Public Involvement (PPI).

Source: [R1, R2, R3, R4, R5, R8, R11, R12, R13, R15, R16, R17, R21]

Candidate Theme 9: RRI & Youth Mental Health

General description: This theme focuses on the application of Responsible Research and Innovation (RRI) principles in the context of youth mental health. It encompasses perceptions of both researchers and young people regarding the need for diverse and meaningful participation in youth mental health projects.

Feature (a): Diverse Participation

Description: This feature highlights the importance of ensuring a broad and inclusive range of young people are involved in youth mental health projects, reflecting the need for representation across various backgrounds and experiences.

Source: [R1, R4, R8, R17, YP4]

Feature (b): Meaningful Participation

Description: This feature focuses on the necessity of engaging young people in ways that are genuinely impactful, ensuring their contributions are valued and integrated into the project outcomes.

Source: [R1, R3, R10, R11, R16, R18, R21, YP2]

Feature (c): Outlining and mitigating risks

Description: This feature addresses the need to clearly identify potential risks in youth mental health projects and implement strategies to mitigate these risks, ensuring the research is conducted safely and ethically.

Source: [R2, R9, R15, R16]
