# Supplementary material for: Exploring Responsible Research and Innovation (RRI) in youth mental health: reflections from researchers and young people
Source: Res Involv Engagem. 2026 Feb 6;12:31. doi: 10.1186/s40900-026-00848-x (PMC12973806; doi:10.1186/s40900-026-00848-x)
Supplement: Supplementary file 4 — Supplementary Material 4: Additional File 4 - Reflexivity [file 40900_2026_848_MOESM4_ESM.pdf]

Additional File 4 – Reflexivity

Article: Exploring Responsible Research and Innovation (RRI) in Adolescent Mental Health: Reflections from Researchers and Young People

Journal: Research Involvement and Engagement

Authors: Josimar Antônio de Alcântara Mendes; Mathijs Lucassen; Sarah Doherty; Ayan Mahamud; Carolyn Ten Holter; Chris Greenhalgh; Ellen Townsend; Marina Jirotka

Josimar Mendes

My commitment to meaningful coproduction and youth participation is grounded in my academic trajectory and professional engagement with children and young people. After completing my PhD in the United Kingdom, I returned to Brazil to take part in a postdoctoral research project that employed youth-led, co-research methodologies. This participatory model embedded youth participation throughout the entire research process, enabling horizontal, trust-based relationships between them and researchers. It deepened my appreciation of dialogic and collaborative processes and affirmed my belief in the transformative power of coproduction. My subsequent work on RRI in youth mental health has further reinforced the importance of frameworks that meaningfully include young people. I view them as rights-holders with creative and transformative potential to advance social justice and systemic change. Moreover, my academic background includes extensive research on the 'principle of the best interests of the child', with a focus on operationalising this principle for professionals working with children and youths, especially in legal and protection services settings. I consistently advocate for research frameworks that elevate the perspectives of young people, acknowledging their

lived experience as a valuable form of knowledge. I approach qualitative research as a socially accountable endeavour that must be rigorous, responsible, and meaningfully shaped by those it aims to serve.
