# Supplementary material for: Exploring Responsible Research and Innovation (RRI) in youth mental health: reflections from researchers and young people
Source: Res Involv Engagem. 2026 Feb 6;12:31. doi: 10.1186/s40900-026-00848-x (PMC12973806; doi:10.1186/s40900-026-00848-x)
Supplement: Supplementary file 5 — Supplementary Material 5: Additional File 5 - GRIPP2 short form [file 40900_2026_848_MOESM5_ESM.pdf]

Additional File 5 – GRIPP2 short form

Article: Exploring Responsible Research and Innovation (RRI) in Adolescent Mental Health: Reflections from Researchers and Young People

Journal: Research Involvement and Engagement

Authors: Josimar Antônio de Alcântara Mendes; Mathijs Lucassen; Sarah Doherty; Ayan Mahamud; Carolyn Ten Holter; Chris Greenhalgh; Ellen Townsend; Marina Jirotka

Section and topic	Item	Reported on page No
**1: Aim**	Report the aim of PPI in the study	Pgs. 13-15, lines 318-334
**2: Methods**	Provide a clear description of the methods used for PPI in the study	Pgs. 13-15, lines 318-334
**3: Study results**	Outcomes—Report the results of PPI in the study, including both positive and negative outcomes	Pgs. 30-32, lines 682-686
**4: Discussion and conclusions**	Outcomes—Comment on the extent to which PPI influenced the study overall. Describe positive and negative effects	Pgs. 30-32, lines 682-686
**5: Reflections/critical perspective**	Comment critically on the study, reflecting on the things that went well and those that did not, so others can learn from this experience	Pgs. 32-40, lines 688-884
